# Association of rs2294008 and rs9297976 Polymorphisms in PSCA Gene with Gastric Cancer Susceptibility in Uzbekistan

**DOI:** 10.5195/cajgh.2016.227

**Published:** 2016-12-13

**Authors:** Shahlo Turdikulova, Dilbar Dalimova, Abror Abdurakhimov, Bekzod Adilov, Sarimbek Navruzov, Abror Yusupbekov, Mirjalol Djuraev, Suleyman Abdujapparov, Dilshod Egamberdiev, Rustam Mukhamedov

**Affiliations:** 1 Institute of Bioorganic Chemistry Academy of Sciences Republic of Uzbekistan;; 2 National Cancer Center of the Ministry of Health of the Republic of Uzbekistan

**Keywords:** Gastric cancer, rs2294008, rs9297976, genotyping, Uzbekistan

## Abstract

**Introduction:**

Genetic factors play an important role in the development of gastric cancer (GC), a prevalent malignancy in Central Asia. Recent studies have shown that single-nucleotide polymorphisms (SNPs) in several genes are associated with increased GC risk, indicating that genetic variation contributes to gastric carcinogenesis. Located on chromosome 8q24.2, the prostate stem cell antigen (PSCA) gene encodes a 123-amino acid glycoprotein related to the cell-proliferation inhibition and cell-death induction activity. SNPs in PSCA gene have been found to be associated with gastric cancer risk in a genome-wide association study, but results were not conclusive. This study aimed to investigate the association between two polymorphic variants of PSCA gene (rs2294008 and rs9297976) and the susceptibility to gastric cancer in Uzbekistan.

**Methods:**

Two hundred sixty eight patients with gastric cancer and a control group of 248 healthy individuals were included in this study. DNA samples isolated from these groups were genotyped using PCR-RFLP method. Comparative analysis of resulting genotypes showed a statistically significant association between CT genotype and gastric cancer (p=0.03, additive model of inheritance, Cochran-Armitage trend test).

**Results:**

Comparative analysis of the distribution of genotypes of rs2976392 polymorphism did not show a statistically significant difference; however, analysis of the distribution of the rs2976392 genotypes in a subgroup of young women revealed a statistically significant (p = 0.04, additive model of inheritance, Cochran-Armitage trend test) increase in the incidence of AA (38%) and AG (56%) genotypes in patients with GC, compared to the controls (20% and 40%).

**Conclusion:**

Our findings support that PSCA rs2294008 and rs9297976 polymorphism may contribute to the susceptibility to gastric cancer. Genotyping of these polymorphisms can potentially be recommended as one of the criteria for identification of high risk groups for gastric cancer development in Uzbekistan.

Gastric cancer (GC) is the fifth most common cancer in the world, with 952,000 new cases diagnosed in 2012.^1^ Although the incidence of gastric cancer has declined in the general population in the US and in many countries around the world,^2^ it remains highly prevalent in Asia as compared to the West. The estimated rate of gastric cancer in Uzbekistan is 18.4% out of all cancer cases^3^ and GC remains a significant cause of cancer morbidity and mortality in Central Asia. Therefore, GC represents one of the key challenges in the development of cancer prevention and control strategy in Uzbekistan.

Carcinogenesis is multi-stage process resulting from both exogenous factors (environmental and lifestyle) and endogenous factors (genetic, hormonal, immune). In Asia, the prevalence of Helicobacter pylori (H. pylori) infection varies markedly in different countries, and is an important etiological factor for the occurrence of gastric adenocarcinoma.^4^ Previous study on the role of nutritional factors associated with GC in urban dwellers of Uzbekistan showed that the most important factors associated with this malignancy were intake of un-boiled water, artesian water, unrefined vegetable oil, margarine, animal fats, dietary salt, and daily consumption of meat-broth.^5^ The link between GC and water consumption may be explained by salt content in drinking water in some regions of Uzbekistan.^6^ Genetic factors also play an important role in the development of gastric cancer (GC). Previously published systematic review suggested that individuals who carry high-risk genetic variants and demonstrate particular dietary habits (such as high consumption of salty foods) may have an increased risk of gastric cancer compared with those who do not carry high-risk genetic variants.^7^ Recent studies have shown that single -nucleotide polymorphisms (SNPs) in several genes are associated with increased GC risk, indicating that genetic variation contributes to gastric carcinogenesis.

The Prostate Stem Cell Antigen (PSCA) gene is located on chromosome 8q24.2 and encodes a 123-amino acid cell surface protein with 30% homology to stem cell antigen type 2 (SCA-2).^8^ PSCA has been reported to be expressed mainly in differentiating cells rather than stem cells.^9^,^10^ PSCA belongs to the Thy-1/Ly-6 family. Members of this family show a remarkable functional diversity ranging from T-cell activation to apoptosis regulation in the nervous system.^11^ PSCA is expressed in the epithelium of several organs, such as prostate, bladder, gallbladder, and stomach. In the gastric epithelium, the main expression sites are the isthmus and neck regions, which contain differentiating cells. The expression of PSCA is downregulated in the gastric tissue with intestinal metaplasia.^9^

The first genome-wide association study (GWAS) on GC, performed in Japanese population, revealed an association between SNP rs2294008 of PSCA and risk of the diffuse type of GC. Substitution of C to T at rs2294008 in the first exon creates a novel translation start site (Met instead of Thr), which leads to the extension of the protein by 9 amino acids which changes the transcriptional activity of the gene.^2^ The association between rs2294008 and GC risk has been replicated in Caucasian and in some Asian populations.^12^–^16^ Two other SNPs (rs9297976 and rs12155758) were also discovered to be associated with GC in Europeans.^13^

To date, an exploration of the association of the PSCA gene SNPs with GC has not been performed in the Central Asia populations. Uzbeks are the ideal group to investigate in this context, as they represent the largest and fastest growing population in Central Asia. Uniquely positioned on the route of the ancient Silk Road, the Uzbek population is a very interesting population to investigate in regard to its cultural, socioeconomic, and genetic diversity. It is remarkable to note that Uzbek population has been formed by admixture of two or more ancestral populations, thus it offers a unique opportunity for studying the interaction between gene polymorphisms, ethnicity specific genetic factors, as well as environmental contributions to disease occurrence.

The epidemiology of gastric cancer subtypes suggests there is a difference in the genetic background between Asian and Caucasian groups. For example “allele flip” between Asian and non-Asian groups is observed, most prominently in polymorphisms of *IL-1B* and *IL-10*.^17^ In addition, an ethnic difference in terms of mutation frequency in *MLH1* (an important gene for DNA mismatch repair that is associated with GC) between Eastern Asians and Western populations have been revealed.^18^

The key aim of this research is to investigate the association between two polymorphic variants of PSCA gene (rs2294008 and rs2976392) and link them to GC susceptibility in Uzbek populations.

## Methods

### Participants

A total of 268 patients (168 males and 100 females) with gastric cancer who underwent surgery in the Internal Medicine department at the National Cancer Center of the Ministry of Health of the Republic of Uzbekistan (NCC MoH UZB) and Tashkent Regional Oncological Dispensary, were recruited from 2011 to 2013. All subjects were unrelated Uzbeks from Tashkent city and various regions of Uzbekistan. All cancer diagnoses were pathologically confirmed. Clinical data and pathological characteristics of patients were collected and confirmed from both their medical records and questionnaire data. The control group constituted of 248 healthy individuals (131 males and 117 females) without a family history of gastric cancer. Cancer patients were recruited through Tashkent Regional Oncological Dispensary. Control cases were obtained from previously implemented study of healthy Uzbekistan people residing in the same catchment area as the majority of our cancer cases.

Before taking part in a study all study participants gave their informed consent. The study was conducted according to the standards of the National Ethic Committee of Uzbekistan developed in accordance with the World Medical Association’s Declaration of Helsinki “Ethical Principles For Medical Research Involving Human Subjects” with amendments (2013) and approved by NCC MoH UZB.

According to the previously published data, the prevalence of GC in young patients ranged from 4.4% to 16.2% of all GC cases when the cut-point was set at 40 years old.^19^,^20^ In light of these studies and also due to the fact that intestinal metaplasia, an aging process due to acid reflux affecting the gastric mucosa, mostly affects patients above the age of 40 years with no significant gender difference,^21^ we also have used this cut-point, when dividing the participants into 4 subgroups. These subgroups were as follows: men older than 40 years (n=139), women over 40 years (n=84), men up to 40 years (n=29), and women up to 40 years of age (n=16).

### Laboratory methods

Blood samples (2 ml) were drawn from an antecubital vein, which were collected using vacutainers containing sodium citrate. Samples were stored at −20°C until they were ready for analysis. Genomic DNA was extracted from peripheral blood leucocytes by using DNA extraction kit Diatom™ DNA Prep 200 (IsoGen Laboratory,^22^ Moscow, Russia). Rs2294008 and rs2976392 polymorphisms were genotyped by means of polymerase chain reaction-restriction fragment length polymorphism (PCR-RFLP). Primer sequences for rs2294008 and rs2976392 were as follows:

Sense 5′-GAAACCCGCTGGTGTTGACTGT-3′ and antisense 5′-GGGCAAGCAGCACAGCCTAC-3′ for rs2294008; sense 5′-ATCTTTCTGGCCATCTGTCCGCAGCT-3′ and antisense 5′-GGCAGATGGACCACCCGCTG-3′ for rs2976392. PCR mixture(25 µl) consisted of 13 µl of ddH2O, 2.5 µl 10xPCR buffer, 2.5 µl 25 mM MgCl_2_, 2.5 µl 2.5 mMdNTP Mix, 1,5 µl (10pkmol/µl) of each oligonucleotide primer, 0.3 µl (1.5 units) “hot-start” Taq-polymerase, and 3 µl of DNA. PCR amplification was carried out in GeneAmp 9700(Applied Biosystems). The PCR conditions were as follows for rs2294008: 95 °C for 5 minutes, and then 33 cycles of 94°C for 45 seconds, 62°C for 30 seconds, and 72 °C for 30 seconds, and a final extension step of 72°C for 5 minutes. PCR conditions for rs2976392 were: 95 °C for 5 minutes, and then 33 cycles of 95°C for 40 seconds, 60°C for 30 seconds, and 72 °C for 40 seconds, and a final extension step of 72°C for 5 minutes. Protocol for implementing these studies has been developed at the laboratory where this study was conducted.

PCR products were digested overnight at 37°C with HpyCH4IV (New England Biolabs) for rs2294008 and PvuII (New England BioLabs) for rs2976392 and then separated by electrophoresis on 3% agarose with ethidium bromide staining and were visualized using WiseDoc WGD-30 (DAIHAN, Korea).

### Statistical analysis

The Hardy-Weinberg equilibrium was tested by a goodness-of-fit χ2 test to compare the observed genotype frequencies with the expected ones among the control subjects. Genotypic associations of SNPs were evaluated by Cochran-Armitage trend test, followed by risk assessment using odds ratio (OR) and 95% confidence of interval (CI) computation. All statistical analyses were performed by using STATA software version 12.0 for Windows (Stata Corporation, USA). A p<0.05 (two-sided) was considered statistically significant.

## Results

**Figure 1:**
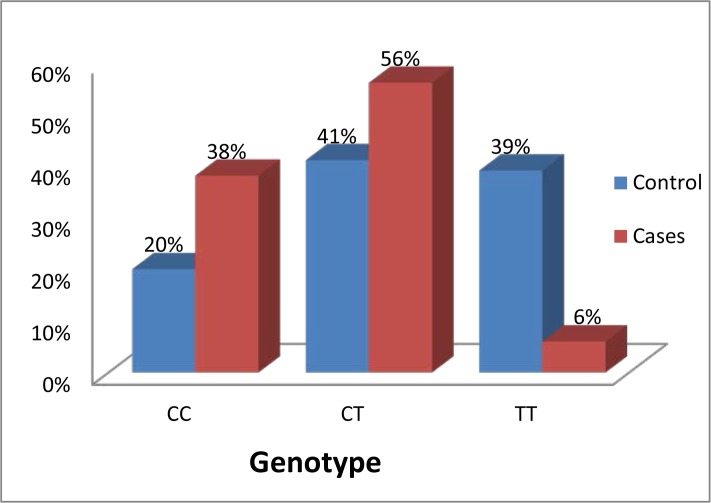
**Genotype distribution of prostate stem cell antigen rs2294008 polymorphism in control group and in subgroup of young women with gastric cancer**

**Figure 2:**
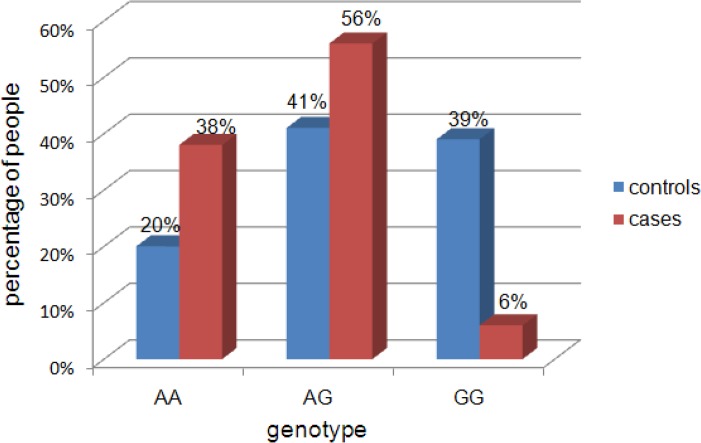
**Genotype distribution of prostate stem cell antigen rs2976392 polymorphism in control group and in patients with gastric cancer (both males and females included)**

Genotype frequencies of PSCA rs2294008 and rs2976392 polymorphisms in patients with gastric cancer and controls are shown in Figures 1 and 2. The genotype distributions of these polymorphisms were in Hardy–Weinberg equilibrium in control groups (p >0.05).

Comparative analysis of rs2294008 genotypes between patients and controls showed a significant association between the CT genotype and gastric cancer (p=0.03, additive model of inheritance, Cochran-Armitage trend test) (see Table 1). The OR of increased relative risk of developing gastric cancer for CT genotype carriers was 3.11 (95% CI: 2.16 – 4.47).

Comparative analysis of the genotype distribution for rs2976392 polymorphism did not show a statistically significant difference between the cases and controls. For the further analysis of rs2976392 genotypes, the group of patients with gastric cancer was divided into 4 subgroups according to gender and age.

Comparative analysis of the distribution of alleles and genotypes of rs2976392 polymorphism did not show a statistically significant difference between subgroups of patients and controls for both men and women of advanced age (over 40 years) and young men (up to 40 years)(see Table 2 and 3). However, analysis of the distribution of the rs2976392 genotypes in subgroup of young women revealed a statistically significant (p=0.04, additive model of inheritance, Cochran-Armitage trend test and p*=*0.03, Fisher’s Exact Test) increase in the incidence of AA (38%) and AG (56%) genotypes in patients with GC, compared to the controls (20% and 41%). The OR of developing gastric cancer for carriers of the AA and AG genotype was 2.35 (95% CI: 0.71–7.76) and 1.88 (95% CI: 0.61–5.72), respectively.

## Discussion

Results of our study confirm the association between the T allele of rs2294008 (CT genotype) with GC risk. Three previous GWAS studies revealed a significant association of the T allele of rs2294008 with the risk of GC^9^,^23^,^24^ and the C allele for duodenal ulcer.^25^ Several case-control studies replicated the association of this SNP with GC in Asian and Caucasian populations.^26^,^27^

The results of the present study also indicated that genetic effect of PSCA rs2976392 polymorphism in Uzbek population is only present in the subgroup of women under the age of 40. This is consistent with the fact that complex genetic diseases, such GC are likely to arise due to multiple, potentially interacting, genetic and environmental factors, and therefore are challenging to study. Presumably, many of these environmental and genetic risk factors are interconnected, with other factors, such as ethnicity, specific genetic background, and endocrine factors, and are likely to be key modifiers of these risk factors. This general phenomenon is known as effect modification, and represents an interaction between two or more variables.

There is some evidence regarding the potential role of estrogen receptors in the regulation of normal development and functioning of the gastric mucosa cells, as well as in the process of tumorogenesis^28^, which provide an intriguing reason to investigate the stomach in relation to estrogen exposure. In this respect, some authors have suggested a close link between receptor mechanism of estrogen action and the growth and proliferation of gastric tumor cells. In one-third of patients with gastric cancer, estrogen receptors have been identified in gastric tumors, suggesting a possible involvement of estrogens in GC development.^29^ At the molecular level, the estrogen receptors function as ligand dependent transcriptional factors that activate or inhibit the expression of target genes in response to hormonal stimulation. It is also interesting to note that estrogen receptors regulate PSCA gene expression, since there is an “ER-binding site” in the promoter region of PSCA gene.^30^ Perhaps this may explain the association of rs2976392 with GC in the subgroup of young women, since they have high levels of estrogen hormones.

It should be noted that there are several limitations of this study. The data concerning subgroup of young women (under the age of 40) with GC may not accurately reflect the prevalence of rs2976392 genotypes in the general population of young women of Uzbekistan because of the small sample size of this subgroup. A large-scale study of young women with GC is needed to evaluate more precisely the contribution of these genotypes to the development of GC in this cohort. Our future studies will evaluate more closely the contribution of Helicobacter Pylori infection to the GC in our cohorts. Overall, considering the burden of GC in Asia, this study is very important for Uzbekistan and the entire Asian continent. In addition, further research on deep sequencing of the PSCA gene is needed to determine the full allelic spectrum of causal variants underlying predisposition to GC and to discover rare variants. It is reasonable to hypothesize that not only the common GWAS variants are responsible for GC risk in Uzbekistan, but also that these loci may contain high effect rare risk variants that have gone undetected by GWAS. It is plausible that future analyses of low-frequency (0.5% ≤ MAF < 5%) and rare (MAF < 0.5%) variants of the PSCA gene using Next-Generation Sequencing (NGS) technologies could explain additional GC disease risk in Uzbekistan.

In summary, the results of our research provided preliminary evidence that PSCA rs2294008 and rs9297976 polymorphism may contribute to gastric cancer risk in Uzbekistan. As studies of this nature are rare in Uzbekistan, this research has important implications for both Uzbekistan, and Central Asian population due to high burden of GC. Genotyping of these polymorphisms can potentially be recommended as a criterion for identification of high risk groups for the development of gastric cancer in Uzbekistan.

## Figures and Tables

**Table 1:** Association between the genotypes of rs2294008 and gastric cancer

Genotype	Controls N (%)	Cases N(%)	Additive model*	OR (95% CI)
CC	119 (48)	78 (29)	χ^2^= 4.97 *p=*0.03	0.45 (0.31 – 0.64)
CT	109 (44)	190 (71)		3.11 (2.16 – 4.47)
TT	20 (8)	0 (0)		0.02 (0.00 – 0.35)

* Using Cochran-Armitage trend test

**Table 2:** Comparative analysis of the distribution of alleles of rs2976392 and gastric cancer

Subgroup	Genotype	Controls N (%)	Cases N (%)	Additive model	OR (95% CI)
Men (>40 yrs)	AA	14 (24)	40 (29)	χ^2^= 0.12 *p=*0.73	1.70 (0.86 – 3.39)
	AG	38 (52)	51 (36)		0.53(0.30 – 0.95)
	GG	21 (24)	48 (35)		1.31(0.71 – 2.42)
Women (>40 yrs)	AA	12 (21)	21 (25)	χ^2^=1.16 *p=*0.28	1.28 (0.57 – 2.86)
	AG	21 (36)	35 (42)		1.26 (0.63 – 2.51)
	GG	25 (43)	28 (33)		0.66 (0.33 – 1.32)
Men (≤40 yrs)	AA	9 (16)	7 (24)	χ^2^=0.11 p=0.74	1.73(0.57 – 5.25)
	AG	31 (53)	12 (41)		0.61(0.25 – 1.51)
	GG	18 (31)	10 (35)		1.17(0.45 – 3.01)
Women (≤40 yrs)	AA	12 (20)	6 (38)	χ^2^= 6.45 p=0.04	2.35 (0.71–7.76)
	AG	24 (41)	9 (56)		1.88(0.61–5.72)
	GG	23 (39)	1 (6)		0.10(0.01–0.84)

* Using Cochran-Armitage trend test

**Table 3 :** Association between the genotype frequencies of rs2976392 and gastric cancer

Subgroup	Genotype	Controls N (%)	Cases N (%)	p-value*
Men (>40 yrs)	AA	14 (24)	40 (29)	*p=*0.09
	AG	38 (52)	51 (36)	
	GG	21 (24)	48 (35)	
Women (>40 yrs)	AA	12 (21)	21 (25)	*p=*0.49
	AG	21 (36)	35 (42)	
	GG	25 (43)	28 (33)	
Men (≤40 yrs)	AA	9 (16)	7 (24)	*p*=0.49
	AG	31 (53)	12 (41)	
	GG	18 (31)	10 (35)	
Women (≤40 yrs)	AA	12 (20)	6 (38)	*p=*0.03
	AG	24 (41)	9 (56)	
	GG	23 (39)	1 (6)	

* Using Fisher’s Exact test
